# High lesion detection rates with preoperative ⁹⁹ᵐTc-PSMA-SPECT/CT prior to radioguided surgery in biochemical recurrence: a hypothesis-generating analysis of quantitative PSMA-PET/CT parameters

**DOI:** 10.1186/s13550-026-01539-0

**Published:** 2026-10-28

**Authors:** Dominic Ufton, Jan Heilinger, David Pfister, Axel Heidenreich, Alexander Drzezga, Matthias Schmidt

**Affiliations:** 1 Department of Nuclear Medicine, University Hospital Cologne, Cologne, Germany; 2 Department of Urology, University Hospital Cologne, Cologne, Germany

## Introduction

Prostate-specific-membrane antigen positron emission tomography with computed tomography (PSMA-PET/CT) has transformed the staging and restaging of prostate cancer, enabling detection of small recurrent lesions amenable to targeted salvage surgery in patients with biochemical recurrence (BCR). ^99m^Tc-PSMA has shown favorable diagnostic performance as an alternative to PET-based imaging in centers with limited PET/CT availability [1], and has been implemented for radioguided surgery (RGS), allowing intraoperative lesion detection via gamma probe combined with preoperative localization via single photon emission computed tomography (SPECT/CT) [2, 3], potentially improving oncologic outcome [4]. Preoperative SPECT/CT serves a dual role in this context: it validates lesion presence in close temporal proximity to surgery and provides the surgeon with anatomical guidance for probe-guided resection. Evidence suggests that RGS contributes to disease control in patients with BCR, with favorable short-term biochemical response rates reported in early cohort studies [5, 6].

However, intermodal agreement between PSMA-PET/CT and ^99m^Tc-PSMA-SPECT/CT remains variable in the literature [7–9], and it is unknown whether quantitative PET/CT parameters — such as maximum standardized uptake value (SUV_max)_ or functional tumor volume (FTV)— can reliably predict whether a given lesion will be detectable on preoperative SPECT/CT. Identifying such a threshold would have direct clinical relevance: patients with sufficiently high PET parameters might be expected to have SPECT-visible lesions with high confidence, potentially allowing selective omission of the preoperative SPECT/CT step and thereby reducing procedural burden and logistical complexity. We investigated this association in a cohort of 30 BCR patients planned for ^99m^Tc-PSMA-RGS.

## Methods

### Patients & imaging

30 patients with BCR and suspect lesions on PSMA-PET/CT underwent intravenous 99mTc-PSMA (MIP-5060, ROTOP; mean activity 740 ± 33 MBq) injection, followed by preoperative SPECT/CT (Symbia Intevo Bold, Siemens Medical Solutions; ordered-subset conjugate gradient reconstruction; low-dose CT 110 kV, 30 mAs; images acquired 4 h post injection.) and RGS the following day. Histopathologic reports were obtained for all resected specimens.

### Statistical analysis

Quantitative PET/CT parameters (SUV_max_, SUV_mean_, FTV [SUV threshold 4.0], short-axis diameter (SAD) in mm) for the single lesion with the highest SUV_max_ on PSMA-PET/CT as well as preoperative prostate specific antigen (PSA) were compared between SPECT-positive (SPECT^pos^) and SPECT-negative (SPECT^neg^) patients using the Mann-Whitney U test with *p* < 0.05 considered significant. Additionally, exploratory receiver operating characteristic (ROC) analysis was performed for SUV_max_, SUV_mean_, and FTV to estimate discriminatory ability for SPECT/CT positivity and to derive AUC values as effect size estimates for future sample size calculations. Given only *n* = 5 SPECT^neg^ patients, all ROC results are explicitly exploratory; no definitive clinical thresholds are derived. A secondary exploratory analysis examined the difference between patients with histologically confirmed metastatic disease (H^+^) and those without confirmed disease (H^−^).

## Results

**Fig. 1:**
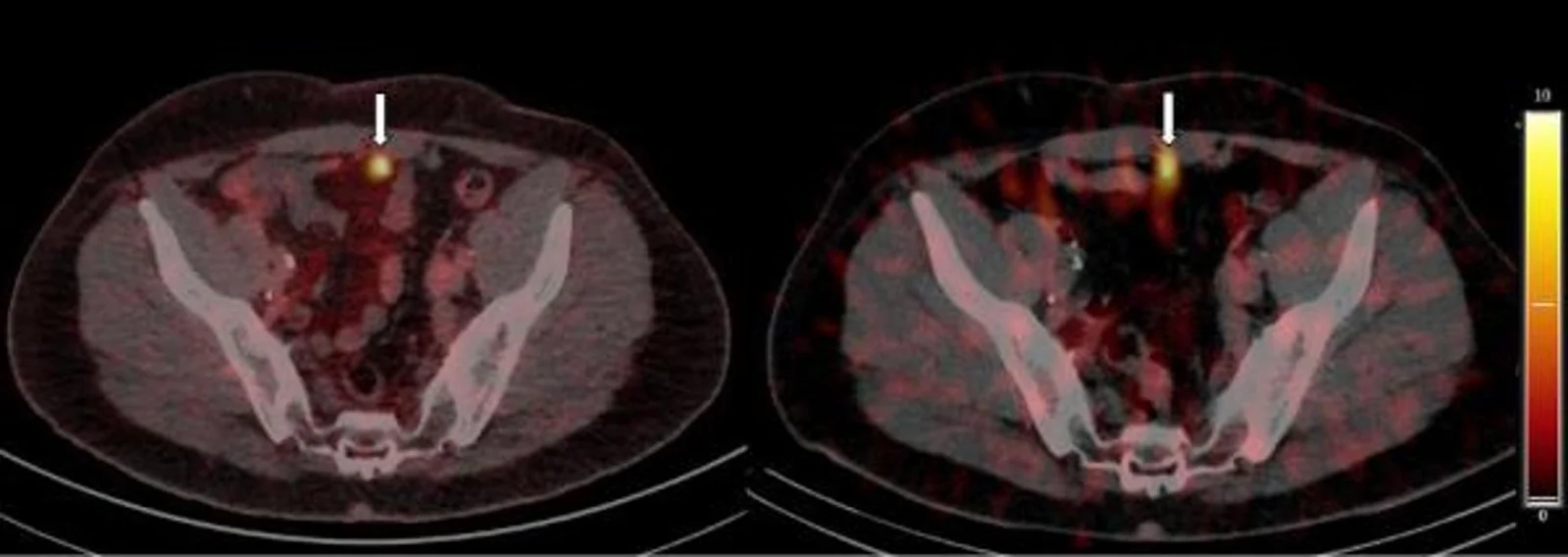
Left: PSMA-PET/CT. Right: ^99m^Tc-PSMA-SPECT/CT 4 h p.i. demonstrates excellent lesion validation of a soft tissue metastasis prior to radioguided surgery (PET/CT SUV_max_ 13.3, SPECT/CT SUV_max_ 11.0)

25/30 patients (83%) showed correlatable lesions on SPECT/CT, an example is given in Fig. 1. All analyzed lesions were located within the pelvic nodal basin. Lesion SAD did not differ significantly between SPECT-positive and SPECT-negative patients (7 ± 2 mm vs. 6 ± 2 mm; *p* = 0.95; Table 1). Average PSA was 1.7 ng/ml ± 1.7 ng/ml. Mean PET/CT parameters across the full cohort were: SUV_max_ 13.6 ± 6.9, SUV_mean_ 6.8 ± 1.7 and FTV 0.9 ± 0.8 ml. As shown in Table 1, all parameters aside from PSA were numerically higher in SPECT_pos_ patients, but no parameter reached statistical significance. On a patient level, histologically confirmed metastatic disease was present in 20/30 patients; SPECT/CT was false-negative in 2/30 patients with confirmed metastases and true-negative in 3/30 histologically negative patients (Table 2). An exploratory subgroup analysis demonstrated markedly higher mean SUV_max_ in SPECT^pos^/H^+^patients compared to those with negative histopathology (17.2 (*n* = 18) vs. 7.6 (*n* = 7); *p* = 0.11).

Exploratory ROC analysis yielded an AUC of 0.71 (95% CI 0.50–0.93, *p* = 0.051) for SUV_max_, 0.67 (95% CI 0.46–0.88, *p* = 0.113) for SUV_mean_, and 0.61 (95% CI 0.35–0.86, *p* = 0.406) for FTV. SUV_max_ demonstrated the strongest trend towards discriminating SPECT^pos^ from SPECT^neg^ patients, but confidence intervals were uniformly wide, reflecting the limited SPECT-negative group. No reliable discrimination threshold could be established from the present data.

**Table 1:** Quantitative PSMA-PET/CT parameters and PSA by SPECT/CT status

Parameter	All (*n* = 30)	SPECT^pos^ (*n* = 25)	SPECT^neg^ (*n* = 5)	*p*-value
SUV_max_	13.6 ± 6.9	14.6 ± 7.7	8.4 ± 2.1	0.15
SUV_mean_	6.8 ± 1.7	7.0 ± 2.0	5.8 ± 0.7	0.25
FTV [ml]	0.9 ± 0.8	1.0 ± 1.0	0.4 ± 0.2	0.47
PSA preoperative [ng/ml]	1.7 ± 1.7	1.6 ± 1.6	2.1 ± 2.4	0.69
SAD [mm]	7 ± 2	7 ± 2	6 ± 2	0.95

Values are presented as mean ± standard deviation

**Table 2:** Cross-tabulation of SPECT/CT status and histopathological confirmation

	SPECT^pos^	SPECT^neg^	Total
**H** ^**+**^	18	2	20
**H** ^**−**^	7	3	10
**Total**	25	5	30

## Discussion

Our oligometastatic cohort of patients with prostate cancer recurrence demonstrated an 83% SPECT/CT detection rate, numerically higher than the 48.3% reported by Rauscher et al. in a comparable patient population using ^111^In-PSMA, however, given differences in tracer, administered activity and SPECT/CT reconstruction algorithms between the two studies, this comparison is indirect and hypothesis-generating rather than a controlled comparison [9]. This may be due to differences in administered activity, the use of ^99m^Tc-PSMA rather than ^111^In-PSMA and advances in SPECT/CT reconstruction algorithms. Furthermore, PSA was comparatively high in our cohort; it should be noted that these PSA values reflect the preoperative clinical context immediately prior to RGS, rather than the initial trigger for PET-imaging.

The directional trend of higher PET parameters in SPECT-positive patients is biologically plausible. SUV in PSMA-PET/CT has been shown to correlate directly with immunohistochemical PSMA expression [10], and it is reasonable to infer that higher PSMA expression equally enhances ^99m^Tc-PSMA uptake, improving lesion delineation on SPECT/CT. The notably higher SUV_max_ in SPECT_pos_/H^+^ patients is directionally consistent with this hypothesis, although the small, non-significant subgroup comparison precludes any firm conclusion. The use of PSF and TOF reconstruction in our PET/CT protocol may have contributed to elevated absolute SUV estimates, particularly for sub-centimeter lesions [11–13]. The directionally higher preoperative PSA in SPECT-negative patients is counter-intuitive, as higher PSA would generally be expected to reflect greater tumor burden and easier lesion detection; however, this comparison is based on only 5 SPECT^neg^ patients and not statistically significant and is likely attributable to small-sample variability rather than a genuine inverse relationship.

Lesion detectability in SPECT/CT may further be enhanced by delayed image acquisition. Berliner et al. demonstrated significantly higher detection rates with late imaging (> 15 h p.i.) compared to standard acquisition at four hours, both on a patient basis (79% vs. 27%) and a lesion basis (60% vs. 21%), with SUV_max_ on prior PSMA-PET/CT and the injection-to-imaging interval identified as independent predictors of SPECT/CT detectability [14]. All patients in the present cohort were imaged at a single, fixed 4-hour time point; our comparatively modest SUV_max_–detectability association may therefore in part reflect this shorter uptake interval rather than a true absence of a dose–response relationship between PSMA expression and SPECT detectability. Because our protocol did not vary in acquisition timing, we could not test this hypothesis directly within our own cohort, and our null ROC finding should accordingly be interpreted as compatible with, rather than contradictory to, the positive association reported by Berliner et al. under a substantially longer uptake interval. Neither Mann-Whitney analysis nor ROC analysis identified a reliable discriminating threshold for SPECT/CT positivity. The AUC of 0.71 for SUV_max_ suggests moderate potential discriminatory value, but the 95% CI (0.50–0.93) spans from chance-level to excellent performance, precluding any clinical application. The principal constraint is the limited number of SPECT-negative patients (*n* = 5); a post-hoc power analysis indicates that approximately 150–200 patients would be required to confirm this effect size with 80–90% power at α = 0.05.

## Limitations

This study was designed to evaluate the association between quantitative PSMA-PET/CT parameters and SPECT/CT lesion detectability and did not include a structured collection of postoperative oncologic outcomes. Intraoperative gamma-probe confirmation data were similarly not captured. Prospective studies linking preoperative SPECT/CT and PET parameters to standardized postoperative biochemical and clinical outcomes are needed to establish the oncologic relevance of the imaging findings reported here. Although patients undergoing androgen-deprivation therapy (ADT) in BCR generally have higher detection rates on PSMA-SPECT/CT, the impact of ADT on RGS is unknown; as it was not systematically recorded in this retrospective cohort it could not be evaluated as a potential confounder of SPECT/CT visibility [15]. There are two further limitations regarding the patient-level analysis. Quantitative PET/CT parameters were derived from a single, highest-SUV_max_ lesion per patient rather than from every individual PET-positive lesion; a lesion-based analysis accounting for within-patient correlation was therefore not conducted. Second, a complete lesion-level histopathological count — the total number of nodes examined and confirmed positive per patient — was not systematically tabulated for this preliminary series and could not be reported.

## Conclusion

In patients with BCR and oligometastatic disease on PSMA-PET/CT planned for ^99m^Tc-PSMA-RGS, preoperative SPECT/CT achieved lesion localisation in 83% of cases, irrespective of quantitative PET/CT parameters. No PET parameter threshold reliably predicted SPECT negativity in this preliminary cohort, and the exploratory ROC analysis confirmed that no threshold can currently justify safe SPECT/CT omission. All patients should undergo preoperative imaging with SPECT/CT until adequately powered prospective studies establish validated thresholds. Future studies should prospectively evaluate delayed SPECT/CT acquisition protocols and their impact on detection rates and surgical outcomes.

## Data Availability

The datasets generated and/or analyzed during the current study are available from the corresponding author on reasonable request.

## Abbreviations

⁹⁹ᵐTc: Technetium-99 m
ADT: Androgen-deprivation therapy
AUC: Area under the curve
BCR: Biochemical recurrence
CI: Confidence interval
CT: Computed tomography
FTV: Functional tumor volume
H+: Histologically positive (confirmed metastases)
H−: Histologically negative
MBq: Megabecquerel
PET: Positron emission tomography
p.i.: Post-injection
PSA: Prostate-specific antigen
PSMA: Prostate-specific membrane antigen
RGS: Radioguided surgery
ROC: Receiver operating characteristic
SAD: Lesion short-axis diameter
SD: Standard deviation
SPECT: Single-photon emission computed tomography
SUV: Standardized uptake value
SUV_max_: Maximum standardized uptake value
SUV_mean_: Mean standardized uptake value

## References

[1] Wang Q, Ketteler S, Bagheri S, Ebrahimifard A, Luster M, Librizzi D. u. a. Diagnostic efficacy of [99mTc]Tc-PSMA SPECT/CT for prostate cancer: a meta-analysis. BMC Cancer 8 August. 2024;24(1):982. 10.1186/s12885-024-12734-4. PMC11312272 39118101

[2] Duin JJ, de Barros HA, Donswijk ML, Schaake EE, van der Sluis TM. Wit EMK, u. a. The Diagnostic Value of the Sentinel Node Procedure to Detect Occult Lymph Node Metastases in PSMA PET/CT Node–Negative Prostate Cancer Patients. J Nuclear Med 1 Oktober. 2023;64(10):1563–6. 10.2967/jnumed.123. .265556 PubMed PMID: 37414445. 37414445

[3] Maurer T, Robu S, Schottelius M, Schwamborn K, Rauscher I, van den Berg. NS, u. a. 99mTechnetium-based Prostate-specific Membrane Antigen–radioguided Surgery in Recurrent Prostate Cancer. Eur Urol 1 April. 2019;75(4):659–66. 10.1016/j.eururo.2018.03.013. 29625755

[4] Bravi CA, Knipper S, Heidenreich A, Fossati N, Gandaglia G, Dell’Oglio P. u. a. Oncologic Outcomes of Template Versus Radioguided Salvage Lymph Node Dissection for Node-only Recurrent Prostate Cancer on Prostate-specific Membrane Antigen Positron Emission Tomography Scan: Results from a Multi-institutional Collaboration. Eur Urol Focus 1 November. 2025;11(6):921–5. 10.1016/j.euf.2025.05.019. 40830006

[5] Su Hchuan, Zhu Y, Hu S, Liu long, Lin C, Dai G et al. B, The Value of 99mTc-PSMA SPECT/CT-Guided Surgery for Identifying and Locating Lymph Node Metastasis in Prostate Cancer Patients. Ann Surg Oncol. 1. Februar 2019;26(2):653–9. 10.1245/s10434-018-6805-y 30324468

[6] Quarta L, Mazzone E, Cannoletta D, Stabile A, Scuderi S, Barletta F. u. a. Defining the optimal target-to-background ratio to identify positive lymph nodes in prostate cancer patients undergoing robot-assisted [99mTc]Tc-PSMA radioguided surgery: updated results and ad interim analyses of a prospective phase II study. Eur J Nucl Med Mol Imaging 1 Oktober. 2024;51(12):3789–98. 10.1007/s00259-024-06789-5. PMC11445289 38861182

[7] Albalooshi B, Al sharhan M, Bagheri F, Miyanath S, Ray B, Muhasin M. u. a. Direct comparison of 99mTc-PSMA SPECT/CT and 68Ga-PSMA PET/CT in patients with prostate cancer. Asia Ocean J Nucl Med Biol. 2020;8(1):1–7. .1293 PubMed PMID: 32064277; PubMed Central PMCID: PMC6994779. 10.22038/aojnmb.2019.43943.1293 PMC6994779 32064277

[8] Fallahi B, Khademi N, Karamzade-Ziarati N, Fard-Esfahani A, Emami-Ardekani A, Farzanefar S. u. a. 99mTc-PSMA SPECT/CT Versus 68Ga-PSMA PET/CT in the Evaluation of Metastatic Prostate Cancer. Clin Nuclear Med Februar. 2021;46(2):e68. 10.1097/RLU.0000000000003410. 33234922

[9] Rauscher I, Maurer T, Souvatzoglou M, Beer AJ, Vag T, Wirtz M. u. a. Intrapatient Comparison of 111In-PSMA I&T SPECT/CT and Hybrid 68Ga-HBED-CC PSMA PET in Patients With Early Recurrent Prostate Cancer. Clin Nuclear Med September. 2016;41(9):e397. 10.1097/RLU.0000000000001273. 27276206

[10] Woythal N, Arsenic R, Kempkensteffen C, Miller K, Janssen JC, Huang K. u. a. Immunohistochemical Validation of PSMA Expression Measured by^68^ Ga-PSMA PET/CT in Primary Prostate Cancer. J Nucl Med Februar. 2018;59(2):238–43. 10.2967/jnumed.117.195172. 28775203

[11] Munk OL, Tolbod LP, Hansen SB, Bogsrud TV. Point-spread function reconstructed PET images of sub-centimeter lesions are not quantitative. EJNMMI Phys 13 Januar. 2017;4:5. 10.1186/s40658-016-0169-9 . PubMed PMID: 28091957; PubMed Central PMCID: PMC5236043. PMC5236043 28091957

[12] Ufton D, Heilinger J, Roth KS, Schmidt M, Drzezga A, Kobe C. A comparison between two image reconstructions on the measures of prostate-specific membrane antigen–positive tumor volume in 18F-PSMA-JK-7-PET/computed tomography. Nuclear Med Commun Oktober. 2025;46(10):967. 10.1097/MNM.0000000000002021. 40551434

[13] Prieto E, Domínguez-Prado I, García-Velloso MJ, Peñuelas I, Richter JÁ, Martí-Climent JM. Impact of Time-of-Flight and Point-Spread-Function in SUV Quantification for Oncological PET. Clin Nuclear Med Februar. 2013;38(2):103. 10.1097/RLU.0b013e318279b9df. 23334123

[14] Berliner C, Steinhelfer L, Chantadisai M, Kroenke M, Koehler D, Pose R. u. a. Delayed Imaging Improves Lesion Detectability in [99mTc]Tc-PSMA-I&S SPECT/CT in Recurrent Prostate Cancer. J Nuclear Med 1 Juli. 2023;64(7):1036–42. 10.2967/jnumed.122. .265252 PubMed PMID: 37230531. 37230531

[15] Li B, Duan L, Shi J, Han Y, Wei W, Cheng X. u. a. Diagnostic performance of 99mTc-HYNIC-PSMA SPECT/CT for biochemically recurrent prostate cancer after radical prostatectomy. Front Oncol 7 Dezember. 2022;12. 10.3389/fonc.2022.1072437. PMC9768541 36568205

